# Proof of Concept of a Gamified DEvelopmental Assessment on an E-Platform (DEEP) Tool to Measure Cognitive Development in Rural Indian Preschool Children

**DOI:** 10.3389/fpsyg.2020.01202

**Published:** 2020-06-10

**Authors:** Debarati Mukherjee, Supriya Bhavnani, Akshay Swaminathan, Deepali Verma, Dhanya Parameshwaran, Gauri Divan, Jayashree Dasgupta, Kamalkant Sharma, Tara C. Thiagarajan, Vikram Patel

**Affiliations:** ^1^Centre for Chronic Conditions and Injuries, Public Health Foundation of India, Gurugram, India; ^2^Child Development Group, Sangath, Goa, India; ^3^Department of Global Health and Social Medicine, Harvard Medical School, Boston, MA, United States; ^4^Sapien Labs, Arlington, VA, United States

**Keywords:** serious game, cognitive development, LMIC, digital assessment, mHealth, machine learning, scalable, preschool children

## Abstract

Over 250 million children in developing countries are at risk of not achieving their developmental potential, and unlikely to receive timely interventions because existing developmental assessments that help identify children who are faltering are prohibitive for use in low resource contexts. To bridge this “detection gap,” we developed a tablet-based, gamified cognitive assessment tool named DEvelopmental assessment on an E-Platform (DEEP), which is feasible for delivery by non-specialists in rural Indian households and acceptable to all end-users. Here we provide proof-of-concept of using a supervised machine learning (ML) approach benchmarked to the Bayley’s Scale of Infant and Toddler Development, 3rd Edition (BSID-III) cognitive scale, to predict a child’s cognitive development using metrics derived from gameplay on DEEP. Two-hundred children aged 34–40 months recruited from rural Haryana, India were concurrently assessed using DEEP and BSID-III. Seventy percent of the sample was used for training the ML algorithms using a 10-fold cross validation approach and ensemble modeling, while 30% was assigned to the “test” dataset to evaluate the algorithm’s accuracy on novel data. Of the 522 features that computationally described children’s performance on DEEP, 31 features which together represented all nine games of DEEP were selected in the final model. The predicted DEEP scores were in good agreement (ICC [2,1] > 0.6) and positively correlated (Pearson’s *r* = 0.67) with BSID-cognitive scores, and model performance metrics were highly comparable between the training and test datasets. Importantly, the mean absolute prediction error was less than three points (<10% error) on a possible range of 31 points on the BSID-cognitive scale in both the training and test datasets. Leveraging the power of ML which allows iterative improvements as more diverse data become available for training, DEEP, pending further validation, holds promise to serve as an acceptable and feasible cognitive assessment tool to bridge the detection gap and support optimum child development.

## Introduction

Nurturing care during early childhood leads to lasting positive impacts, including more grades completed in school, and higher adult incomes (Alderman et al., 2017; Nandi et al., 2017; Trevarthen et al., 2018), thereby forming the foundations to achieving the Sustainable Development Goals (Daelmans et al., 2017). However, using proxy measures of poverty and stunting which are known to reflect poor brain development, recent estimates indicate that nearly 250 million children in low and middle-income countries (LMICs) below 5 years of age, of which 65 million live in India, fail to attain their full developmental potential (Lu et al., 2016). These proxy measures are likely to underestimate the true extent of this burden since they are not direct measures of brain functioning. Using a more direct measure – the Early Child Development Index (ECDI) – one study suggested that 81 million children in the age group of 3–4 years alone were developing sub-optimally across 35 LMICs, with sub-Saharan Africa and South Asia contributing the largest numbers (McCoy et al., 2016).

While these statistics are alarming, a growing body of evidence suggests that early interventions targeted to optimize development can mitigate the impact of adversities, increase resilience, and protect developmental trajectories (Jeong et al., 2018). However, routine developmental assessments which aid in timely identification of children in need of interventions are rarely conducted in LMICs because they are heavily dependent on skilled healthcare professionals conducting lengthy assessments using expensive and proprietary tools. This confluence of adverse environments and expensive, resource intensive developmental assessments leads to large “detection” gaps, whereby children with developmental impairments remain unidentified and underserved (Dasgupta et al., 2016).

Therefore, efforts are underway to develop scalable and cross-culturally valid tools for assessment of early childhood development (ECD), so that children in need of interventions receive timely referrals. Although significant progress has been made, it is still an emerging science with key limitations. First, the ECD field tends to focus largely on the first 1000 days (Elmadfa and Meyer, 2012; Ghosh, 2016; Wrottesley et al., 2016). However, brain development continues beyond the first three years, making it imperative to extend developmental monitoring to at least until the time when children start formal schooling where systems are in place to regularly assess each child through metrics of academic performance. Second, existing tools are limited by either being based on (1) parent report, the accuracy of which is often dependent on the parent’s knowledge of child development and reliance on crude developmental milestones, or (2) assessor observation, which requires extensive training and regular supervision to build assessor skills.

These limitations have led to a growing need for ECD assessments that are objective and accurate, as well as feasible for delivery by non-specialists requiring minimal training and supervision. One solution could be the use of mobile technology which has demonstrated immense potential in scaling up services in low resource settings. m-Health strategies are increasingly being used as job-aids by community health workers (CHWs) in LMICs. A systematic review demonstrated that the use of technology not only empowered and motivated them but also improved their credibility to the communities they served (Agarwal et al., 2015). CHWs empowered with mobile data collection tools are also more efficient and the data is less prone to errors compared to paper-pencil tools (Thies et al., 2012). Additionally, children from the age of 2 years on have been shown to interact meaningfully with touch-screen tablets, understand rules of playing digital games and provide appropriate responses through gestures such as taps and drags, providing a unique opportunity to use gamified neuropsychological tasks for directly assessing children’s developmental abilities (Semmelmann et al., 2016). The emerging literature demonstrates that computerized neuropsychological assessments are reliable and valid (Martinovic et al., 2015), and the introduction of “gamification” increases participant engagement and ecological validity (Lumsden et al., 2016; Pitchford and Outhwaite, 2016). The advantages of data in digital format, beyond improvements in efficiency and accuracy, can be further complemented by the use of advanced analytics such as machine learning, which has the potential to predict outcomes using a data-driven approach (Anzulewicz et al., 2016; Bosl et al., 2018), and continually update algorithms to iteratively improve the accuracy of these predictions as more relevant data become available. Therefore, m-Health technology is not only acceptable to all end users, but has all the other ingredients necessary to create a validated, cross-cultural, and scalable tool.

Although computerized versions of classical neuropsychological tests (CANTAB, CogState) are already available (De Luca et al., 2003; Williams et al., 2016), they are cost-intensive and typically not designed for very young children (2–4 years). Other low-cost, open-source tools have similar limitations since they tend to focus on academic skills such as reading and math (Hubber et al., 2016; Miller, 2018; Pitchford et al., 2018), which are unsuitable for assessing preschool children who are yet to develop literacy-numeracy skills, thereby missing the window of opportunity of the early years when the brain is maximally plastic. The limited number of tools that are usable in the preschool age-range typically focus only on one or a few domains of cognition such as attention and memory (McPherson and Burns, 2008; Vergauwe et al., 2009).

These gaps call for the development of a new digital assessment tool which allows for comprehensive assessment of a diverse range of cognitive skills in preschool children. Therefore, our interdisciplinary team created “DEvelopmental Assessment on an E-Platform” (DEEP) to fill this gap (Bhavnani et al., 2019). DEEP is a gamified cognitive assessment tool comprising age-appropriate games administered on Android tablets. The DEEP games were designed in collaboration with an expert team comprising a developmental pediatrician, psychiatrist, clinical psychologist, neuroscientists, machine learning experts and game developers through consensus workshops, and tap into multiple cognitive skills including manual processing speed, manual coordination, attention, response inhibition, reasoning, visual form perception, visual integration, and memory. An initial prototype was developed, which underwent multiple rounds of iteratively testing to improve the acceptability, feasibility and ease of administration of DEEP in our study setting. Pilot testing of the current version of DEEP on 3 years old children in rural Indian settings found it to be (1) highly acceptable to children and their families, (2) feasible for delivery by non-specialists in rural households, and (3) capable of discriminating children’s cognitive abilities based on the variability of performance on the games (Bhavnani et al., 2019).

In this study, we explored the potential of DEEP to measure cognitive development of 3 years old children. To this end, we used a supervised machine learning approach to predict a child’s Bayley’s Scale of Infant and Toddler Development, 3rd Edition (BSID-III) cognitive score, using metrics derived from gameplay on DEEP. We chose to benchmark DEEP to BSID-III in this pilot study because it is the most widely used research tool to measure child development globally (Tran et al., 2014; Springer et al., 2018), including in India (Balakrishnan et al., 2018; Thomas et al., 2019), thereby enabling comparisons of our cohort with other studies. BSID-III assesses development across five domains including cognition, language and motor and is suitable for children aged 1–42 months. However, as highlighted for existing standardized tools, BSID-III is costly, time-intensive and requires high levels of assessor skills and training, making it prohibitive for use at scale in low resource contexts. Given our mandate to develop alternative scalable options, we asked two critical questions of DEEP – (1) how accurately can a child’s performance on DEEP predict their BSID-III cognitive score, and (2) how accurately can DEEP identify children who score below the 25th percentile on the BSID-III cognitive assessment. Once validated, DEEP will help in identifying children with delayed or impaired cognitive abilities or children with a neurodevelopmental disorder which impacts cognitive functioning.

## Materials and Methods

### Study Site and Participants

The participants in this cross-sectional study were recruited from 120 villages in Rewari district in rural Haryana, India, and comprised 200 children (51.5% girls) aged 34–40 months randomly selected from the SPRING ECD trial cohort (Lingam et al., 2014; Divan et al., 2015; ClinicalTrials.gov, 2017). Bhopal et al. (2019) has previously reported cohort and study site details. The exclusion criteria while recruiting the target sample size of 200 were (1) vision and hearing loss or impairment as reported by the parent, (2) any other condition that impeded the child from interacting meaningfully with the tablet computer, or (3) refusal of parental consent. Prior to data collection, the objectives and methods of our study were explained to the parent and written informed consent was obtained from those who agreed to participate in this study. Appointments were scheduled as per the family’s convenience and data was collected from January–October 2018. This study was conducted in accordance with the Declaration of Helsinki and approved by the institutional ethics committees of the Public Health Foundation of India and Sangath.

### Study Tools

The following tools were administered by a team of 2 non-specialists (henceforth referred to as assessors) through household visits conducted over 1.5 h each on 2 consecutive days:

#### DEEP Questionnaire

A short parent-report questionnaire was administered to obtain information on the child’s attendance in private or government preschools, prior exposure to smartphones and digital games, and overall health and well-being on the day of the assessment.

#### Bayley’s Scale of Infant and Toddler Development, 3rd Edition (BSID-III)

A translated version of the BSID-III (Albers and Grieve, 2007) adapted for administration by non-specialists was used following a protocol described previously (Bhopal et al., 2019). Bhopal et al. demonstrated that BSID-III scores in all domains of development (cognitive, language, and motor) at 18 months of age were negatively associated with all measures of childhood adversity in this cohort, as would be expected from the scientific literature on the impact of adversities on ECD (Hair et al., 2015; Luby, 2015; Pavlakis et al., 2015), and providing validation to this version of the BSID-III in our study site. The BSID-III assessment for 3 year olds was delivered by the same outcome assessment team as in the SPRING RCT study (ClinicalTrials.gov: SPRING Cluster Randomized Controlled Trial; Lingam et al., 2014; Divan et al., 2015). These assessors were rigorously trained and supervised by ECD specialists, with inter-rater reliability between the assessors being greater than 99%. BSID-III is an observation based tool and involves a series of tasks for a child to complete including object manipulation, demonstrating understanding of basic concepts (color, shape, size, numbers, etc.), and simple physical activity. Six BSID-III sub-scales were administered – cognitive, receptive and expressive language, fine and gross motor and social-emotional. Any child unable to meet BSID-III milestones appropriate for 25.5–28.5 months was referred to pediatric clinics for follow-up assessments. The raw, scaled, and composite scores were calculated following protocols described in the manual.

#### DEEP Gamified Assessment

DEEP is a gamified cognitive assessment tool comprising age-appropriate games administered on Android tablets (Samsung Tab E), and takes about 20–30 min to complete (Bhavnani et al., 2019; Supplementary Figure S6). It has nine games, each with 2–6 levels of difficulty, woven together through a first person narrative. DEEP games tap into multiple cognitive skills including manual processing speed, manual coordination, hand-eye coordination, attention, response inhibition, reasoning, visual form perception, visual integration, and memory (Bhavnani et al., 2019). At the beginning of each game, the assessor delivers verbal instructions during a demo-mode, where the child is taught how to play the games and allowed to practice, with help from the assessor if required. In cases where children are not able to follow verbal instructions or imitate the tap or drag movements of the assessor on the tablet, the assessor holds the child’s index finger to guide him/her on how to make the appropriate gesture (tap or drag) in order to play the game. The assessor is taught to proceed to play mode if the child can play correctly without assistance from the assessor (during the demo mode). Child performance is only recorded in the backend during the play-mode.

#### Anthropometry

The assessors used World Health Organization (WHO) protocols (WHO, 2012) to measure a child’s height and weight and Centers for Disease Control and Prevention (CDC) protocol to measure head-circumference using the Seca 213 Portable Stadiometer, SECA-384 electronic scale, and Seca 201 Mechanical measuring tape respectively. Stunting and underweight were defined as two standard deviations below the age-adjusted median values of height and weight respectively as per WHO standards. All children whose anthropometric measurements were below three standard deviations of WHO age-adjusted median values were referred for follow-up assessments.

### Data Analysis

#### Outcome Variable

The raw BSID-III score of the cognitive subscale, treated as a continuous variable and henceforth referred to as *BSID-cognitive score*, was used as the outcome variable to train the machine learning models.

#### Predictor Variables: Feature Set Derived From DEEP Backend Data

Meaningful features that tap into a wide range of cognitive skills were extracted from the DEEP backend (see Bhavnani et al., 2019) for a description of the cognitive domains assessed by DEEP games and Supplementary Table S1 for a description of the types of features computed from the DEEP backend). Feature extraction was done in consultation with experts (developmental pediatrician, clinical psychologist, and neuroscientists) to ensure that each feature taps into cognitive skills. For example, the feature type “accuracy” in the game “matching shapes,” where a child needs to drag an object to its matching shadow, taps into multiple cognitive skills such as visual form perception, inhibitory control, attention, planning etc., while the feature type “latency” in the same game taps into another set of cognitive skills such as processing speed and attention. It is important to note that all the included features tap into multiple cognitive skills, and likewise, each cognitive skill is assessed by multiple features across all the nine games of DEEP.

262 features comprising the number and timestamp of correct, incorrect and background taps and drags were extracted as raw data (see Table 2; features from the tablet). These features were used to compute 709 additional derived features such as accuracy, playtime and activity (Supplementary Table S1), resulting in a total of 971 features across nine games (Table 2). Derived features were computed for (1) each level of a game, (2) all levels of a game combined, and subsequently (3) for a combination of all nine games (represented in Table 2 as “Across games”). Missing data for each level or game that a child was unable to attempt was replaced with meaningful values. For example, accuracy for missed levels was assigned 0 and completion time was assigned the maximum time allowed to complete that level (game timer). For a complete list of assigned values, please refer to Supplementary Table S7. Histograms were generated for each feature to evaluate the distribution of the data. Features with skew values > 1 or < -1 were transformed using square-root and square functions respectively for the data to more closely approximate a normal distribution. Highly correlated features (Pearson’s *r* > 0.9) were dropped to avoid multi-collinearity while training the models, leading to an initial set of 412 uncorrelated features for exploratory analysis.

Since our feature set was extensive, adding interaction terms derived from the entire dataset would have been computationally unwieldy. Therefore, interaction terms were derived only from a smaller subset of 20 features (Supplementary Table S2) that were selected into the top models during an initial exploratory ML run as two-way products and ratios of features in this subset. Only viable (those having < 15% null values) and uncorrelated features (those with Pearson’s *r* < 0.9 with the earlier feature set) were retained, resulting in 83 interaction terms being added. One feature was engineered using the mas-o-menos (mom) algorithm (Zhao et al., 2014). Finally, the first 26 principle components, which explained 70% of the variance in the dataset were added. Therefore, the final feature set comprised 412 (initial feature set) + 83 (interaction terms) + 1 (mas-o-menos) + 26 (principle components) = 522 features (see Table 2 for a description of the number of features contributed by each game and additional derivations). The dataset was scaled for all subsequent steps.

#### Brief Description of the Machine Learning (ML) Algorithm

The ML analysis was run using the R statistical software version 3.5.2 (R Core Team, 2014). The total sample of 200 children was randomly split with 70% (*N* = 140) contributing to training the models (Training set), and the remaining 30% (*N* = 60) being assigned to a “test set” which was kept naïve to the training protocol and only used to determine model performance (accuracy and generalizability) of the final algorithm on a novel dataset.

**FIGURE 1:**
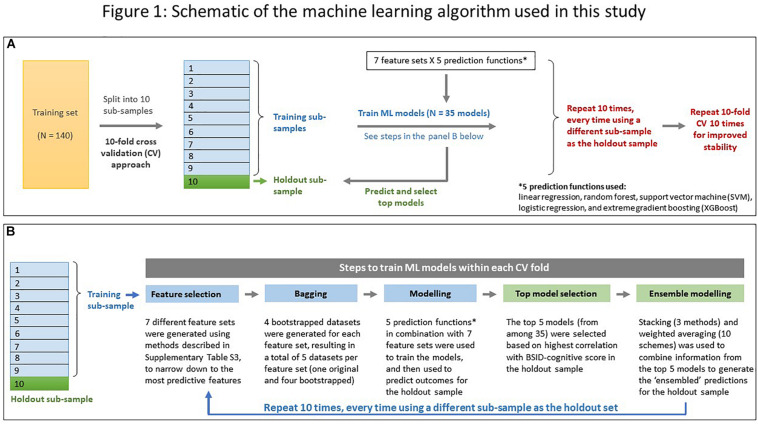
Schematic of the machine learning algorithm used in this study. **(A)** A 10-fold cross validation (CV) approach was used. The training dataset (*N* = 140) was randomly split into ten “folds.” During each CV run, data from ninefold were combined to form the training subsample while the remaining fold was used as the holdout sample. Operations in the training sub-sample are coded in blue, and those in the holdout sample in green. The 10-fold CV was repeated 10 times for improved stability. **(B)** Details of the five steps employed in each CV run while predicting a child’s cognitive score. Color coding same as in **(A)**.

Seven different feature selection methods were applied to select a smaller subset (capped at 15 features per set) that comprised the best predictors of the BSID-cognitive score (Supplementary Table S3). These were used in combination with five prediction functions (linear regression, random forest, support vector machine, extreme gradient boosting, and logistic regression) to train the ML models using a 10-fold cross-validation (CV) approach (see Figure 1 for a schematic of the ML approach used), repeated ten times to improve stability. Predictions from the top 5 models, based on the highest correlation with the BSID-cognitive score, were combined using ensemble modeling (stacking and weighted averaging) to derive the final prediction score for each child (henceforth referred to as the “DEEP” score). The top five models were chosen for ensembling since it minimized the bias-variance trade-off across 5 ML runs (Supplementary Figure S4). For a detailed description of the individual steps used in our ML approach, please refer to Figure 1 and Supplementary Material.

#### Model Performance Metrics

The primary goal of DEEP is to measure a child’s cognitive development, hence we first focused on the accuracy of our algorithm to predict the continuous BSID-cognitive score. The distributions of DEEP and BSID-cognitive scores in the training and test datasets were compared by plotting histograms and the agreement between the two scores was visualized using Bland-Altman plots. Model performance was quantified using (1) intra-class correlation coefficient using two-way random effects model – ICC(2,1) – with 95% confidence interval, (2) mean prediction error – defined as the average difference (DEEP – BSID) between the two scores, (3) mean *absolute* prediction error and (4) root-mean-squared-error (RMSE). Pearson’ correlation coefficient (r) with 95% confidence interval was used to report the correlation between DEEP and BSID-cognitive scores. The strength of agreement and correlation based on ICC(2,1) and Pearson’s r was assessed as per criteria defined in the literature (Swinscow, 1997; Li et al., 2015).

Additionally, to determine the discriminating ability of DEEP to identify children with poor cognitive abilities (defined here as scores below the 25th percentile on the BSID-cognitive scale in our sample), receiver operating characteristics (ROC) curve was drawn. The (1) sensitivity (true positive rate), (2) specificity (true negative rate), (3) area under the curve (AUC), and (4) accuracy (% correct classification in the whole dataset) were tabulated for both the training and test datasets.

#### Impact of Prior Exposure to Digital Games and Fine Motor Skills on DEEP Predictions

Our data indicated that no child in our sample had ever played games or interacted with tablet computers before our visit, but 57% (*N* = 113) of them had experience playing games on a smartphone. We hypothesized that children experienced in playing games on touchscreen devices would perform better on the DEEP assessment, which could bias DEEP’s accuracy in predicting a child’s BSID-cognitive score. We tested our hypothesis by comparing the prediction errors for these two groups of children. The significance of the difference in means was tested using Student’s unpaired *t*-test with equal variance at the α = 0.05 level.

We also hypothesized that children with better fine motor skills (as measured by the fine-motor subscale of the BSID-III) would perform better on DEEP given the requirement of hand-eye coordination and the use of drag-and drop gestures. We tested this by plotting the prediction errors against the BSID-III fine motor domain scores and determined the strength of the correlation using Pearson’s r (with 95% confidence interval). Additionally, to determine the proportion of variance of children’s fine motor skills that was explained by DEEP’s prediction errors, the *R*^2^-value was examined.

## Results

### Description of Study Participants

The socio-demographic and developmental profile of the study participants is summarized in Table 1, stratified by their presence in the training or test datasets. No significant differences were observed for any of the measures between the two groups. Although not statistically significant, a higher proportion of children in the training dataset attended government preschools – the Anganwadi centers in India. A third of the children were stunted and over a quarter were underweight as per WHO norms. Since children in our sample were spread across three age brackets as per the BSID-III manual, the age-adjusted composite scores in the cognitive, motor and language sub-scales are reported to summarize the developmental attainment level of this sample. The mean BSID-III cognitive composite score (US standardized norms) in the training dataset was 89.29 [95% CI: 70.02–108.55] which matched closely with the test set (89.00 [95% CI: 72.19 -105.81]). The mean BSID-III motor and language composite scores were also comparable across both the groups, indicating that the overall developmental status of the two groups of children were similar.

**TABLE 1:** Socio-demographic and developmental profile of study participants.

Study participant profile	Training set (*N* = 140)	Test set (*N* = 60)	*p-*value^$^
Mean age in months (SD)	37.25 (0.86)	37.47 (0.68)	0.06
% Male	48.57	48.33	0.98
Mean height in cm (95% CI)	90.23 (83.07–97.38)	90.47 (83.14–97.79)	0.67
Stunting (%)^#^	31.65	30	0.84
Mean weight in kg (95% CI)	11.87 (9.10–14.65)	11.97 (9.10–14.84)	0.67
Underweight (%)^#^	28.57	26.67	0.78
Mean head circumference in cm (95% CI)	47.51 (43.87–51.15)	47.54 (44.84–50.23)	0.91
Preschool enrollment (%)			
Not attending	47.14	60	0.06
Private	31.43	31.67	
Anganwadi centers*	21.43	8.33	
Mean BSID-III cognitive composite score (95% CI)	89.29 (70.02–108.55)	89.00 (72.19–105.81)	0.84
Mean BSID-III motor composite score (95% CI)	104.47 (79.01–129.93)	101.67 (77.46–125.87)	0.15
Mean BSID-III language composite score (95% CI)	97.72 (78.67–116.77)	96.57 (76.27–116.86)	0.46

^#^Stunting and underweight have been defined as per World Health Organization benchmarks of measures below two standard deviations of the median Height for Age z-score (HAZ) and Weight for Age z-score (WAZ) respectively. *Government preschools in India. ^$^p-value is based on the X ^2^ test for differences in proportions among categorical variables tabulated against presence in the training or test datasets, or based on Students’ t-test for continuous variables.

### Predicting the BSID-III Cognitive Score Using Backend Data From DEEP

#### Pattern of Incomplete Gameplays on DEEP

Once children engaged with the first few games, they generally went on to attempt all the other games. Of the 200 children, 95.5% attempted all nine games and only 4.5% were incomplete (did not attempt at least one game). Only two children did not attempt five or more games. These two children were also the lowest BSID-III scorers in the cohort and both received referrals for further developmental evaluations based on their BSID-III performance.

#### Models Used to Predict the DEEP Score

The top five models (defined here as those generating predictions with the highest correlations with BSID-cognitive scores) selected during each run of the cross-validation folds were tabulated in a frequency table to record the best performing models across ten repeats of 10-fold CV. The prediction functions most commonly selected to predict the BSID-cognitive score from the DEEP metrics were extreme gradient boosting (XGBoost) and support vector machine (Supplementary Table S4). The DEEP score for each child was derived by weighted averaging of the three stacked predictions using a weighting scheme of 0.25, 0.25, and 0.50 for linear regression, random forest and XGBoost stacking functions respectively (see Figure 1, Supplementary Figure S5, and Supplementary Table S5).

Of the 522 features that computationally described children’s performance on DEEP (Table 2), 31 unique features were selected in the final model (Table 3). Except for one feature that was directly extracted from the DEEP backend (latency in level 1 of matching shapes), all other features were higher order derivations of the raw data (*n* = 30; examples include accuracy, activity, interaction terms and mas-o-menos; Table 3). Eight out of the 11 types of features computed from the DEEP backend (Supplementary Table S1) were represented in the final feature set.

**TABLE 2:** Feature set extracted from the DEEP backend metrics and selected in the final model.

Game	Features from the tablet	Derived features	Total	Feature set used for ML modeling^#^	Selected in final model
Single tap	3	6	9	4	–
Alternate tap	4	12	16	8	–
Popping Balloons	6	30	36	11	–
Grow your garden	30	90	120	40	–
Hidden objects	37	119	156	86	–
Odd one out	74	166	240	105	–
Matching shapes	27	59	86	13	1
Jigsaw puzzles	32	66	98	24	3
Location recall	49	131	180	111	–

Across games	0	30	30	10	4
Interaction terms^$^	–	–	–	83	22
Principle components*	–	–	–	26	–
Mas-o-menos	–	–	–	1	1
**Total**	**262**	**709**	**971**	**522**	**31**

^#^Highly correlated features (Pearson’s r > 0.9) were dropped to avoid multicollinearity during modeling, resulting in 522 features being used for training the machine learning models from the initial set of 971 features. ^$^Interaction terms were generated by computing products and ratios of a subset of the features selected from the initial exploratory analysis (see Supplementary Table S2). *26 principle components explained 70% of the variance in the dataset.

**TABLE 3:** Features selected in the final prediction model.

Sr. #	Feature source	Features selected in the final prediction model*	Feature type^#^	Game
1	Individual games	jig_av_correctrate_sqrt	Derived	Jigsaw (JIG)
2		jig_l5_accuracy_cbyi_sqrt	Derived	
3		jig_l5_activity	Derived	
4		ms_l1_latency	Tablet	Matching shapes (MS)
5	Across games	sum_all_levels_played	Derived	All nine games (Across games)
6		sum_completion_time	Derived	
7		sum_total_accuracy_cbyt	Derived	
8		msjig_total_incorrectdrag_sqrt,	Derived	MS + JIG
9	Interaction terms	msjig_total_playtime_times_jig_l1_activity	Derived	MS + JIG
10		jig_l1_activity_times_jig_l3_accuracy_cbyi_sqrt	Derived	JIG
11		jig_l1_activity_times_ms_l2_correctrate_sqrt	Derived	MS + JIG
12		ms_av_playtime_times_jig_l1_activity	Derived	MS + JIG
13		ms_av_playtime_times_jig_l3_accuracy_cbyi_sqrt	Derived	MS + JIG
14		ms_av_playtime_times_ms_l1_accuracy_cbyi	Derived	MS
15		ms_l1_accuracy_cbyi_times_ms_l3_correctrate_sqrt	Derived	MS
16		ms_l1_activity_sqrt_div_by_sum_total_accuracy_cbyt	Derived	MS + Across games
17		ms_l1_activity_sqrt_times_st_correctclicks	Derived	MS + Single tap
18		ms_l1_correctdrags_div_by_sum_total_accuracy_cbyt	Derived	MS + Across games
19		ms_l1_correctdrags_times_ms_l1_totaldrags_sqrt	Derived	MS
20		ms_l1_correctrate_sqrt_div_by_sum_all_levels_played	Derived	MS + Across games
21		ms_l1_correctrate_sqrt_times_ms_l1_accuracy_cbyi	Derived	MS
22		ms_l1_latency_div_by_sum_all_levels_played	Derived	MS + Across games
23		ms_l1_latency_div_by_sum_total_accuracy_cbyt	Derived	MS + Across games
24		ms_l1_latency_times_ms_l1_correctdrags	Derived	MS
25		ms_l1_totaldrags_sqrt_times_st_correctclicks	Derived	MS + Single tap
26		ms_total_incorrectdrag_sqrt_times_jig_l1_activity	Derived	MS + JIG
27		st_correctclicks_times_sum_all_levels_played	Derived	Single tap + Across games
28		msjig_total_incorrectdrag_sqrt_div_by_sum_total_accuracy_cbyt	Derived	MS + JIG + Across games
29		msjig_total_incorrectdrag_sqrt_times_st_correctclicks	Derived	MS + JIG + Single tap
30		msjig_total_playtime_div_by_sum_total_accuracy_cbyt	Derived	MS + JIG + Across games
31	Mas-o-menos	mom	Derived	Across games

*The prefix indicates the game that contributed the feature. Jig, jigsaw puzzles; ms, matching shapes; msjig, combination of matching shapes and jigsaw; sum, across all nine DEEP games; st, single tap. ^#^Tablet, extracted directly from DEEP backend; Derived, higher order derivations of tablet-derived metrics.

Features from two games (matching shapes and jigsaw) dominated, contributing 26 of 31 (83.8%) features in the final feature set, individually or as one of the features used to compute interaction terms (Table 3). 12 of 31 (32.4%) features represented all nine games, including the total time taken to complete the full suite of games and sum of levels played across all games, implicating that the final feature set taps into a wide range of cognitive skills as assessed by all the DEEP games. The feature generated using the mas-o-menos algorithm was also selected into the final model.

#### Agreement Between DEEP and BSID-Cognitive Scores

**FIGURE 2:**
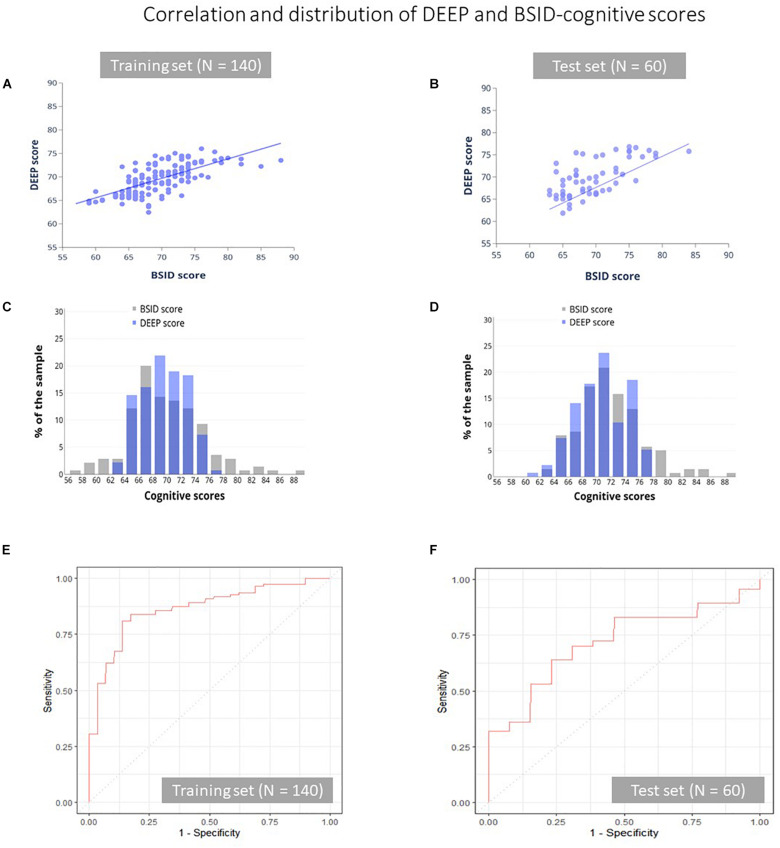
Correlation and distribution of DEEP and BSID-cognitive scores. The correlation between DEEP and BSID-cognitive scores **(A,B)**, their distribution **(C,D)** and the ROC curves of the training **(E)** and test **(F)** datasets is shown. Gray and purple bars represent the BSID-cognitive and DEEP scores respectively.

Our ML algorithm could generate predictions for 195/200 children across the training and test datasets, since five children did not play one or more games that contributed features to the final prediction algorithm. Of these five children, three received a referral for developmental delay based on their low BSID-III scores. We observed moderate positive correlation and good agreement between DEEP and BSID-cognitive scores (Pearson’s correlation coefficient = 0.67, and ICC(2,1) ≥ 0.60 in both the training and test datasets, Table 4 and Figures 2A,B). The mean and standard deviation (in parentheses) of the absolute prediction error was 2.87 (2.36) and 2.88 (2.21) for the training and test datasets respectively (see Table 4 for a comprehensive list of other model performance metrics). There were only two children for whom the prediction error was more than 10 points. Interestingly, these children were the top two BSID scorers and constitutes the group least at risk of developmental impairments. Importantly, model performance of the training and test datasets were highly comparable, indicating high generalizability of our model to novel datasets.

**TABLE 4:** Model performance metrics.

Model performance metrics	Training set (*N* = 137)^&^	Test set (*N* = 58)
Pearson’s correlation coefficient	0.67 (0.57–0.76)	0.67 (0.49–0.79)
ICC(2,1) [95% CI]*	0.604 (0.49–0.70)	0.66 (0.49–0.79)
Mean absolute error (SD)	2.87 (2.36)	2.88 (2.21)
Root mean square error (SD)	3.71 (5.09)	3.62 (4.30)
Mean bias error (SD)	−0.05 (3.72)	0.13 (3.65)
**Receiver Operating Characteristics**		
Sensitivity (true positive rate)	0.846	0.692
Specificity (true negative rate)	0.810	0.697
AUC	0.849	0.721
Accuracy^#^ (%)	83.21	70.69

^&^N refers to the number of children for whom DEEP predictions could be generated. Full dataset: N = 140 (Training set) and N = 60 (Test set). *Agreement levels for ICC(2,1): >0.6 = good. ^#^DEEP cut-off score that optimized accuracy for correct classification (performance above or below the 25th percentile BSID-III cognitive score) was 67.19. The accuracy of the test set predictions was based on this cut-off value.

Although the mean prediction error was low, we observed that DEEP tended to overestimate low BSID scores and underestimated high scores (Supplementary Figure S1). We examined if the poorer predictions at the two ends of the BSID distribution may be due to floor or ceiling effects of the DEEP games (low scorers unable to cross a minimum threshold and high scorers maxing out on all levels), which may have impacted DEEP’s ability to parse out the variation of children’s cognitive abilities at the extremes. We plotted the number of difficulty levels children with low (<25th percentile) and high (>90^th percentile^) BSID-cognitive scores attempted on the DEEP games, with the assumption that a lack of variability would indicate floor and/or ceiling effects. The maximum number of levels a child can play on DEEP is 40. Among the low BSID performers (*N* = 42), only 3 (7.1%) played < 10 levels, while among the high scorers (*N* = 21), 17 (80.9%) were unable to attempt all levels (Supplementary Figure S2). Therefore, floor and ceiling effects were not evident in our sample. Therefore, we hypothesized that poorer predictions at the extreme ends of the BSID distribution was due to the small sample sizes at the tails, which negatively impact model performance (see Figures 2C,D and Supplementary Table S6 that further illustrates the difference in sample sizes between the extremes vs. the middle of the BSID distribution). As a consequence, the range of DEEP scores was lower than BSID-cognitive scores in both the training (DEEP: 62.4–76; BSID: 57–88) and test datasets (DEEP: 61.85–76.8, BSID: 63–84, Figures 2C,D). The implications are discussed later.

#### Discriminating Ability of DEEP to Identify Poor BSID Performers

The 25th percentile BSID-cognitive score (66 in our sample) was used as a cut-off to draw ROC curves, to examine DEEP’s ability to identify poor BSID performers. The overall accuracy of correct classification was 83.2% in the training set and 70.7% in the test set. The area under the curve (AUC) for the training and test datasets were 0.85 and 0.72 respectively (Table 4 and Figures 2E,F). The sensitivity (true positive rate) and specificity (true negative rate) of DEEP for the training set was 0.85 and 0.81 respectively using a cut-off score of 67.62 (which maximized the sum of sensitivity and specificity for the training set). For the test set, the corresponding values were 0.69 and 0.70.

#### Impact of Prior Smartphone Exposure and Fine Motor Abilities on DEEP Validity

**FIGURE 3:**
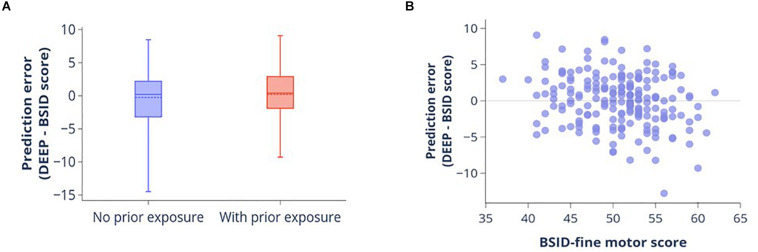
Impact of prior experience of playing digital games and fine motor skills on DEEP’s prediction accuracy. **(A)** Boxplot showing the distribution of prediction errors (DEEP-BSID-cognitive score) for children with and without prior exposure to playing games on a smart device. Mean (dotted line) and median (solid line) are shown. There is no significant difference in mean prediction error between the two groups. **(B)** Scatter plot of prediction error against fine motor score measured by the BSID-III fine motor domain subscale.

Of the 200 children in the combined dataset, 87 (43.5%) had no prior experience of playing games on a touchscreen device. Nonetheless, we observed no significant difference in prediction errors (DEEP-BSID scores) between the children with and without prior exposure (Student’s *t*-test *p*-value = 0.28, Figure 3A). Therefore, we concluded that prior exposure did not impact the quality of DEEP’s predictions.

Similarly, to determine the impact of fine motor skills on prediction accuracy, we evaluated the correlation between BSID-III fine motor score and DEEP’s prediction error. Fine motor skills were very poorly correlated with the prediction error (Pearson’s *r* = -0.25 [95% confidence interval = -0.37 to -0.11], Figure 3B), with the scatterplot showing a random distribution around the mean. Additionally, prediction errors could only explain 6% of the variance of fine-motor skills in our sample.

## Discussion

We recently reported the development and piloting of a gamified cognitive assessment tool named DEvelopmental assessment on an E-Platform (DEEP) (Bhavnani et al., 2019), and demonstrated it to be feasible for delivery by non-specialists in rural Indian households and acceptable to children and their families. In this study we explored the potential of using a supervised machine learning (ML) approach benchmarked to the “gold standard” BSID-III cognitive score, to predict a child’s cognitive development using the backend metrics of DEEP. We found that the predicted DEEP scores were in good agreement with the BSID-cognitive score, with satisfactory ROC metrics.

An important characteristic of our analysis was the inclusion of a “test” dataset to evaluate the accuracy of our algorithm on a novel dataset that did not contribute to training the models. We found that model performance was comparable between the training and test datasets, highlighting the generalizability of our algorithm. Importantly, using a cutoff score of 67.62 that optimized the sensitivity and specificity of DEEP for the training set, the sensitivity of the test set was 0.692 and specificity was 0.667, underscoring the extent of DEEP’s potential in reducing the “detection” gap in our study setting, a prime example of a region where developmental assessments are far from routine.

Contrary to expectations, DEEP’s predictions of a child’s BSID-cognitive score was not impacted by prior experience of playing digital games on touch-screen devices, nor correlated with fine motor skills, presumably due to the ease of play engineered during DEEP’s design, as well as the addition of a “demo” phase which allowed children to practice playing the games before data was collected in the backend.

Two games – matching shapes and jigsaw – contributed the bulk of the features selected in the final model, indicating that cognitive skills related to picture matching and completing jigsaw puzzles are particularly suited to predicting children’s cognitive abilities in our 3 years old, rural North-Indian cohort, although other features representing the entirety of the DEEP games were also selected in the final model. It is important to note here that a few items on the BSID-III cognitive scale for this age range also require matching by shape, color and size, as well as completing simple jigsaw puzzles, highlighting the clinical relevance of features selected in our final model. Some of the cognitive abilities tested by these games include visual form perception, visual integration, attention and response inhibition.

Although our initial results are very encouraging, they highlight a few limitations that need refinement in future versions. One key limitation is the poorer prediction accuracy at the extreme ends of the BSID-III distribution, which we speculate may be due to the small sample size in the tails. For example, of the 200 children in our study sample, only four scored below 60 and five above 80 on the BSID-III cognitive assessment. Therefore, our algorithm performed relatively poorly in predicting these extreme scores compared to predictions in the middle of the BSID-III distribution where the sample size is >100. Consequently, the range of DEEP scores and thereby its sensitivity, is lower than the BSID-III measure. We plan to overcome this issue by enriching our sample at both ends of the BSID-III distribution in future studies to achieve good model performance across the whole spectrum of BSID scores. Another limitation was the inability to predict scores for five children (of the total sample of 200) who did not play key games that contributed features to the final prediction algorithm. However, it is important to note that three of those five children received referrals for follow-up assessments based on their poor BSID-III performance. Therefore, the inability to engage with the DEEP games may be an important indicator of developmental delays warranting further evaluations. We will also follow up this hypothesis in future studies.

Additionally, since DEEP was tested on a very homogenous population (as evidenced in Table 1), it is likely that the current model may perform sub-optimally in other diverse settings and age ranges. Therefore, next steps include (1) administering DEEP on a diverse sample across India and abroad; (2) adding more difficulty levels to allow longitudinal monitoring of children across the preschool years (2–6 years); and (3) integrate other games and modes of assessments (such as eye-tracking) to expand its functionality to assess other developmental domains such as fine motor, social-emotional and language. Given the power of iterative improvements that a machine learning approach allows as more relevant data become available to train the models, we are optimistic that our proposed ways forward would continue to improve the accuracy and generalizability of DEEP.

The need for ECD interventions have been emphasized in LMICs, however, there is a paucity of validated and scalable direct child measures for evaluating the outcomes of these interventions. While stunting may currently be the best proxy measure for human, social and economic capital (Hoddinott et al., 2013), it may be too distal a measure to detect improvements in cognitive abilities, especially if the intervention is unrelated to nutrition (e.g., – parenting support programs improve cognitive development, but have no impact on child growth) (Britto et al., 2017). Given the granularity of the data captured by DEEP, it could provide a possible solution once it has been tested for its sensitivity to detect neurodevelopmental changes brought about by ECD interventions.

Machine learning approaches which allow for the analysis of all available data in an unbiased manner have the potential to identify novel biomarkers of child development. Integral to using the ML approach is feature engineering, which creates complex combinations of available features to improve predictions. In the case of DEEP, the superior predictive capacities of engineered features are demonstrated through the fact that the majority of features selected in our final model are “interaction terms,” and one derived using the mas-o-menos algorithm, all of which constitute novel biomarkers of cognitive development in our cohort.

## Conclusion

In conclusion, it is essential to set up mechanisms wherein children undergo regular monitoring to ensure that they are developing optimally, and refer those who are faltering to effective interventions. In low resource settings, where more than 40% of the children are at risk of not achieving their developmental potential, the absence of scalable assessment tools that can be used by frontline health workers leads to a large detection gap. DEEP, an acceptable and feasible gamified digital tool for assessment of cognitive development (Bhavnani et al., 2019), has now been demonstrated to accurately predict a child’s cognitive development. Leveraging the power of machine learning analytics, we plan to iteratively improve DEEP’s predictions by continuing to collect large samples of diverse data across settings, populations and age groups, as well as study its sensitivity to measure change brought about by ECD interventions. Through these efforts, we hope to create a tool to longitudinally track cognitive development across the preschool years, analogous to the WHO growth standards that monitor physical growth in children, as well as contribute to the dimensional assessment of cognitive development in the early years, aligned with the principles of the Research Domain Criteria (RDoC) framework.

## Data Availability Statement

The datasets generated for this study are available on request to the corresponding author.

## Ethics Statement

This study involving human participants was reviewed and approved by the Institutional Ethics Committees of the Public Health Foundation of India and Sangath. Written informed consent to participate in this study was provided by the child’s primary caregiver.

## Author Contributions

DM, SB, JD, GD, TT, and VP were responsible for study conception and design. DM, SB, KS, and DV were responsible for the acquisition and management of data. DM, SB, AS, DP, TT, and VP analyzed and interpreted the data. DM, SB, AS, and VP drafted the manuscript. All authors edited and approved the manuscript.

## Conflict of Interest

TT, a collaborator from Sapien Labs in her scientific capacity, also holds the position of Chairperson of Madura Microfinance Ltd. The remaining authors declare that the research was conducted in the absence of any commercial or financial relationships that could be construed as a potential conflict of interest.
