# Supplementary material for: Proof of Concept of a Gamified DEvelopmental Assessment on an E-Platform (DEEP) Tool to Measure Cognitive Development in Rural Indian Preschool Children
Source: Front Psychol. 2020 Jun 10;11:1202. doi: 10.3389/fpsyg.2020.01202 (PMC7299081; doi:10.3389/fpsyg.2020.01202)
Supplement: Supplementary file 1 [file Data_Sheet_1.pdf]

Supplementary Material

Exploring the potential of a gamified DEvelopmental assessment on an E-Platform (DEEP) tool to measure cognitive development in rural Indian preschool children

Debarati Mukherjee[1], Supriya Bhavnani[1,3], Akshay Swaminathan[2], Deepali Verma[3], Dhanya Parameshwaran[4], Gauri Divan[3], Jayashree Dasgupta[3], Kamalkant Sharma[3], Tara C. Thiagarajan[4], Vikram Patel[1,2,3*]

[1]Centre for Chronic Conditions and Injuries, Public Health Foundation of India, Gurugram, India
[2]Department of Global Health and Social Medicine, Harvard Medical School, Boston, MA, USA
[3]Child Development Group, Sangath, Goa, India
[4]Sapien Labs, Arlington, VA, USA

*** Correspondence:**
Vikram Patel
vikram_patel@hms.harvard.edu

This supplementary appendix contains a detailed description of the machine learning (ML) approach used in this study to predict a child's cognitive development using backend metrics of DEEP

Feature Set

Meaningful features that measure a diverse set of cognitive skills and comprehensively characterize a child's performance in each game were extracted or computed from DEEP's backend metrics. Supplementary Table S1 lists the source, names, and descriptions of these features. Raw features extracted directly from the backend of DEEP and higher-order derivations of them yielded a total of 971 features across all nine games (Table 2). Features with distributions that were either left- or right-skewed (with skewness values > +1 or < -1 respectively) were replaced with appropriate mathematical transformations (square-root and square) for the data to better approximate a normal distribution. Highly correlated features (Pearson's r > 0.9) were removed to avoid multi-collinearity during model generation, resulting in an initial set of 412 features (out of the 971 features) for exploratory analysis.

We conducted a preliminary run of our machine learning algorithm using these 412 features, which identified a subset of 20 unique features that were the best predictors of the outcome (BSID-cognitive score), in lieu of being selected into the top five models (see Supplementary Table S2 for details). Seventeen of the 20 selected features represented two games – matching shapes and jigsaw, while two of them represented all nine games (sum of all the levels played and the total accuracy across all games).

Generating interaction terms for the entire dataset was impractical due to the large size of the initial feature set, therefore, two-way interaction terms were only generated for the 20 features described above. Interaction terms were computed as products and ratios of each feature with every other feature. Only viable features, those having null values for < 15% of the sample, and uncorrelated (Pearson's r ≤ 0.9) with the original dataset was retained, resulting in 83 interaction terms being added to the original feature set.

One feature was engineered using the mas-o-menos algorithm[1] and added to the feature set. Briefly, in this algorithm, the feature set is scaled and the marginal association of each feature with the BSID-cognitive score is computed. The normalized features, weighted by the signs of their marginal associations, are summed to generate the mas-o-menos score for every child.

Finally, principle components (PC) were computed for the entire feature set and the first 26 PCs that accounted for 70% of the variance in the data were added. Therefore, the final ML algorithm was run on 412 (initial feature set) + 83 (interaction terms) + 1 (mas-o-menos) + 26 (principle components) = 522 features (Table 2 in the main manuscript lists the number of features contributed by each game, as well as each of the above feature types described above). The feature set was scaled prior to training the ML models.

Generating the training and test datasets

The full dataset (N = 200) was randomly split such that 70% of the data comprised the training set (N = 140) and the remaining 30% assigned to the test set (N = 60). ML models were only trained on the training set, and the final algorithm was applied to the test set to evaluate the generalizability and accuracy of the models to predict a naïve dataset that did not contribute to

training the models.

Cross Validation (CV)

We employed 10-fold CV to train our ML models. The training set (N = 140) was randomly split into 10 sub-samples or 'folds' (see Fig. 1A). During each CV iteration, models were trained on data from nine of the 10 folds, and predictions were generated for the remaining hold-out sample. The modelling steps within each CV fold comprised (1) feature selection, (2) bagging, (3) modelling, (4) top model selection, and (5) ensemble modelling, each of which is described below and schematically represented in Fig. 1B.

Feature Selection

We applied seven feature selection methods (see Supplementary Table S3) on the CV training sub-sample (comprising 522 features) to narrow down the feature set to those that were the most predictive. Feature sets were capped to have at most 15 features in order to prevent overfitting (which was about 10% of the number of observations in the training sub-sample).

Bagging

Due to the small sample size of the training set, we employed bagging (bootstrap aggregation) to decrease the variance of the predictions. Bagging improves model stability and accuracy by averaging predictions across several bootstrapped datasets.[2] For each feature set, four bootstrapped datasets were generated by sampling from the CV training sub-sample observations (with replacement), resulting in a total of 5 datasets (1 original + 4 bootstrapped). The optimal number of bootstrapped datasets was determined using cross-validation. For a schematic of the bagging method used in this study, see Supplementary Fig. S3.

Each of 5 prediction functions (linear regression, random forest[3], support vector machine[4], logistic regression, and extreme gradient boosting[5]) in combination with each of the 7 feature sets (Supplementary Table S3) was trained on the 5 datasets, and used to predict the outcomes for the hold-out sample. This resulted in 175 prediction vectors (5 prediction functions X 7 feature sets X 5 datasets) for each child in the holdout sample. Predictions from the five datasets for each model was averaged, to arrive at the final 'bagged's prediction vector for each of the 35 models in the holdout sample (see Supplementary Fig. S3).

Notes about prediction functions

Before performing linear regression, highly correlated features (r > 0.99) were removed. Before running random forest models, missing data were imputed using the "rfImpute" function from the "randomForest" R package.[6] The optimal number of trees set to grow for the random forest was determined through CV and set to 800. For support vector machine models, the linear kernel function was used, and the optimal cost and epsilon parameters were determined through CV and were set to 1 and 0.01 respectively. Before performing logistic regression, the continuous

outcome was converted to a binary outcome using the 25th percentile BSID-cognitive score as the cut-off (66 in our sample). For extreme gradient boosting, the "xgboost" function was used from the "xgboost" package. The optimal maximum number of boosting iterations was determined through cross validation and set to 5, and the "booster" parameter was set to "gblinear".

Top Model Selection

The top models were selected based on the highest pairwise Pearson's correlation co-efficient (Pearson's r) between the 35 bagged prediction vectors corresponding to the 35 models, and the outcome variable (BSID-cognitive score) in the holdout sample.

Ensemble Modelling

The optimum number of top models to use for ensemble modelling was empirically determined by running five iterations of the 10-fold CV using different number of models for ensemble modelling. The root-mean-squared error (RMSE) of the final predictions across the five runs were plotted as boxplots, along with the raw data points (Supplementary Fig. S4). We observed that ensembling the top five models would result in the best bias-variance trade-off, defined as having the lowest RMSE and variance when compared to ensembling the top 2, 3, 4, 6, or 7 top models. The top five models during each CV run were identified and saved for later use.

We used stacking (stacked generalization)[8] and weighted averaging to combine predictions from the top five models. Briefly, stacking involves combining multiple "base learners" via a single meta-learner. The base learners are the top five models selected as described above. Predictions of these top five models comprise the input features for the meta-learner. We stacked predictions of the top five models using 3 different meta-learners: linear regression, random forest and extreme gradient boosting, and used these three models to generate three stacked predictions for the holdout sample.[8] The three stacked predictions were combined using ten different weighted averaging schemes (see Supplementary Table S5 for details on weighting schemes used), which resulted in the generation of 10 prediction vectors for each child in the holdout sample. At the end of the 10-fold CV run, when these 10 'ensembled' prediction vectors were generated for the entire training set (N = 140), the weighting scheme that corresponded to the prediction vector with the least RMSE was identified as the best scheme for the particular CV run (see Supplementary Fig. S5 for a schematic of the ensemble modelling approach used in this study).

Improving Stability

Given the small size of our training set, the results from CV were affected by the particular random allocation of the 140 observations into the 10 CV folds. We therefore repeated the 10-fold CV approach ten times for improved stability. Each run of the 10-fold CV resulted in a slightly different set of top five models being selected, and correspondingly different 'best' weighting schemes. Therefore, to generate predictions for the test set, the final top five models were the five most commonly selected models across the (10 folds per CV iteration) x (10 iterations) = 100 CV folds. Supplementary Table S4 lists the 35 models used in this study sorted based on the number of times they were selected across the 100 folds, with the top five models

that appeared most frequently marked **in bold.** The final weighting scheme was the most commonly selected weighting scheme across the 10 CV iterations (weights for linear regression, random forest and XGBoost = 0.25, 0.25, 0.50 respectively, and marked in **bold** in Supplementary Table S5. This weighting scheme was selected five times across the 10 CV repeats).

References

1. Zhao SD, Parmigiani G, Huttenhower C, Waldron L. Mas-o-menos: a simple sign averaging method for discrimination in genomic data analysis. *Bioinformatics* 2014; **30**(21): 3062-9.
2. Breiman L. Bagging Predictors. *Machine Learning* 1996; **24**: 123-40.
3. Liaw A; Wiener M. Classification and Regression by randomForest. *R News* 2002; **2**(3).
4. Vandewalle JAKSJ. Least Squares Support Vector Machine Classifiers. *Neural Processing Letters* 1999.
5. Chen THT. xgboost: eXtreme Gradient Boosting, 2019.
6. Breiman LC, Adele. Breiman and Cutler's Random Forests for Classification and Regression. CRAN; 2018. p. Classification and regression based on a forest of trees using random inputs, based on Breiman (2001) <DOI:10.1023/A:1010933404324>.
7. Hastie TT, R; Friedman, J. The Elements of Statistical Learning: Springer; 2017.
8. Wolpert D. Stacked generalization. *Neural Networks* 1992; **5**(2): 241-59.

Supplementary Table S1: Types of features extracted or computed from the DEEP backend data

Sr. No.	**Source**	**Feature name**	**Description**
1	Features from the tablet	Latency (Reaction time)	Time taken for the first tap or drag since the start of the game
2		Completion time	Total time taken to complete a level or game
3		Highest level reached	The highest level of difficulty reached in a game
4		# of demo trials played	# of demo trials practiced before switching to game mode
5		# of correct clicks or drags	Number of correct attempts in a level or game
6		# of incorrect clicks or drags	Number of incorrect attempts in a level or game
7		# of background clicks	Number of taps in the background in a level or game
8	Derived	Accuracy	Proportion of correct attempts to total number of attempts or the number of incorrect attempts
9		Rate of correct clicks	# of correct taps per second
10		Rate of incorrect clicks	# of incorrect taps per second
11		Proportion of responses inhibited	# of incorrect responses actively avoided in a response inhibition game
12		Activity	Total number of correct or incorrect attempts divided by completion time
13		Playtime	Completion time minus latency (duration for which the child actively played the game)
14		Inaccuracy	Proportion of incorrect or background taps/drags to total number of attempts

Supplementary Table S2: Features used to derive interaction terms

Sr. No.	**Features selected from initial exploratory ML run and used to generate interaction terms**
1	ms_l1_latency
2	ms_l1_activity_sqrt
3	msjig_total_incorrectdrag_sqrt
4	ms_l1_correctdrags
5	ms_total_incorrectdrag_sqrt
6	msjig_total_playtime
7	sum_total_accuracy_cbyt
8	ms_l1_totaldrags_sqrt
9	st_correctclicks
10	ms_l1_correctrate_sqrt
11	sum_all_levels_played
12	ms_av_playtime
13	ms_l2_latency
14	jig_l1_activity
15	ms_l2_correctrate_sqrt
16	ms_l1_accuracy_cbyi
17	ms_l2_activity_sqrt
18	jig_l3_accuracy_cbyi_sqrt
19	jig_av_correctrate_sqrt
20	ms_l3_correctrate_sqrt

Features selected into the top five models during the initial exploratory ML run were shortlisted, and interaction terms (defined as two-way products and ratios of each feature with every other feature) were derived from this subset of 20 features.
Prefixes refer to the game that contributed the feature: ms = matching shapes; jig = jigsaw; msjig = combination of ms and jig; sum = across all nine games; st = single tap (manual processing speed)

Supplementary Table S3: Description of feature selection methods

**Feature Set Name**	**Method used to derive feature set**
Feature Set 1	Conduct univariate linear regression of BSID-COG with all features. Keep the top 15 features with the lowest Bonferroni-adjusted p-value
Feature Set 2	Intersection between Feature Set 1 and the top 30 features with the largest coefficients in absolute magnitude
Feature Set 3	Convert continuous outcome to binary based on 25th percentile BSID-cognitive score cutoff. Conduct univariate logistic regression with all features. Keep the top 15 features with the lowest Bonferroni-adjusted p-value
Feature Set 4	Intersection between Feature Set 3 and the top 30 features with the largest coefficients in absolute magnitude
Feature Set 5	Convert continuous outcome to binary based on 25th percentile BSID-cognitive score cutoff. Conduct separate two-sample t-tests with all features. Keep the top 15 features with the lowest Bonferroni-adjusted p-value
Feature Set 6	Intersection between Feature Set 5 and the top 50 features with greatest fold change across classes
Feature Set 7	Compute pairwise Pearson correlation coefficient between BSID-cognitive score and all features. Keep the top 15 features with the greatest magnitude of Pearson's r.

Supplementary Table S4: Frequency of models selected among the top five across ten repeats of 10-fold cross-validation

**Sr. #**	**Feature set-model combination**	**Frequency of being among the top 5 models across 10 repeats of 10-fold CV**
**1**	**Feature Set 1_XGBoost**	**38**
**2**	**Feature Set 2_XGBoost**	**31**
**3**	**Feature Set 3_XGBoost**	**31**
**4**	**Feature Set 7_XGBoost**	**30**
**5**	**Feature Set 2_SVM**	**27**
6	Feature Set 5_Random Forest	25
7	Feature Set 3_Random Forest	23
8	Feature Set 1_Linear Regression	22
9	Feature Set 4_Random Forest	20
10	Feature Set 2_Random Forest	18
11	Feature Set 4_Logistic Regression	17
12	Feature Set 5_Logistic Regression	16
13	Feature Set 2_Linear Regression	16
14	Feature Set 5_XGBoost	15
15	Feature Set 1_SVM	15
16	Feature Set 3_Linear Regression	15
17	Feature Set 1_Random Forest	13
18	Feature Set 4_XGBoost	13
19	Feature Set 7_Random Forest	13
20	Feature Set 7_SVM	12
21	Feature Set 3_Logistic Regression	11
22	Feature Set 5_Linear Regression	10
23	Feature Set 4_SVM	10
24	Feature Set 4_Linear Regression	10
25	Feature Set 3_SVM	10
26	Feature Set 7_Logistic Regression	10
27	Feature Set 7_Linear Regression	10
28	Feature Set 5_SVM	9
29	Feature Set 2_Logistic Regression	5
30	Feature Set 1_Logistic Regression	4
31	Feature Set 6_Logistic Regression	1

Top five models used to predict the test set is marked in bold. They appeared most frequently across 100 iterations of the CV runs. Extreme gradient boosting (XGBoost) was the most common prediction function in the top five models. Feature set 2 (see Supplementary Table S3) appeared 2 out of 5 times.

Supplementary Table S5: Weighting schemes used to combine stacked predictions

Weighting scheme #	Weight for Linear Regression	Weight for Random Forest	Weight for Extreme Gradient Boosting
**1**	**0.25**	**0.25**	**0.50**
2	0.25	0.50	0.25
3	0.50	0.25	0.25
4	0.33	0.33	0.33
5	0.50	0.50	0
6	0	0.50	0.50
7	0.50	0	0.50
8	1	0	0
9	0	0	1
10	0	1	0

The weighting scheme selected most frequently (5 of 10 times) across 10 repeats of CV is marked in bold and corresponds to 0.25, 0.25 and 0.50 for linear regression, Random Forest and XGBoost respectively.

Supplementary Table S6:The relationship between sample size and prediction accuracies across the BSID-cognitive score distribution

**BSID-cognitive score (Training dataset, N = 140)**	**N**	**Mean Absolute Error (SD)**
56 – 65 (< 25th percentile)	29	3.16 (2.00)
66 – 75 (25-90th percentile)	97	2.31 (1.65)
76 – 90 (> 90th percentile)	14	6.23 (3.97)

Supplementary Figure S1: Prediction error as a function of BSID-cognitive domain raw score

The Bland-Altman plot shows the scatter of prediction errors (DEEP-BSID score) as a function of the BSID-III cognitive score. DEEP tends to overestimate low scores and underestimate high BSID scores.

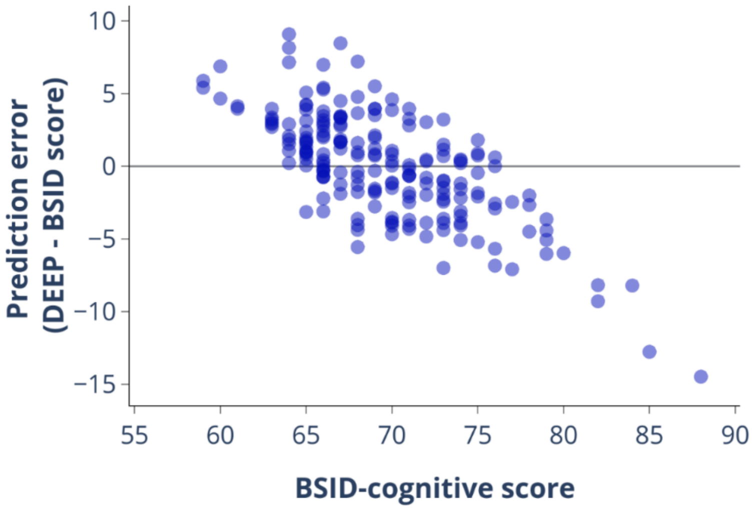

Supplementary Figure S2: Number of difficulty levels attempted by low and high BSID scorers on the cognitive scale

The boxplot demonstrates the variability of the total number of difficulty levels attempted across the nine games of DEEP by two groups of children – those scoring ≤25th percentile (**low scorers**; N = 42) or > 90th percentile (**high scorers**; N = 21) on the BSID-cognitive subscale. Solid lines represent the median value. Dotted line represents the mean. Floor and ceiling effects are not evident.

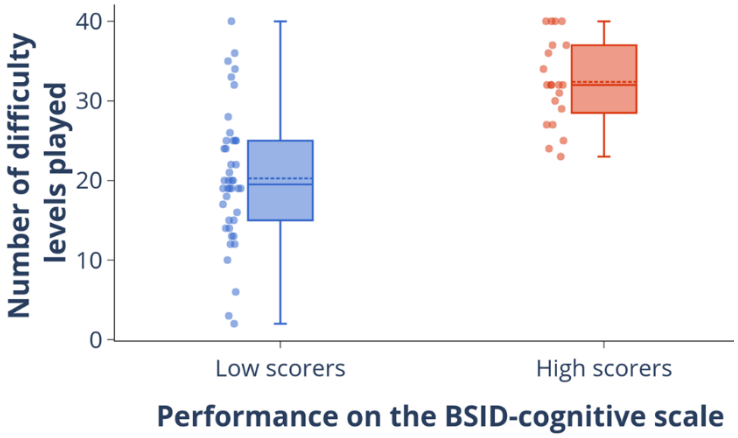

frontiers

Supplementary Figure S3: Schematic of the **b**ootstrapped **ag**gregation (bagging) method used in this study

The four bootstrapped datasets derived from the original training sub-sample is represented as blue rectangles. A combination of 7 feature sets and 5 prediction functions (35 models) were run on all 5 datasets (4 bagged and 1 original), and used to predict the holdout sample. Predictions from the 5 datasets for each model were averaged to result in one 'bagged' prediction vector per model. The top five bagged predictions (based on the highest correlations with the BSID-cognitive score) was later used for ensemble modelling.

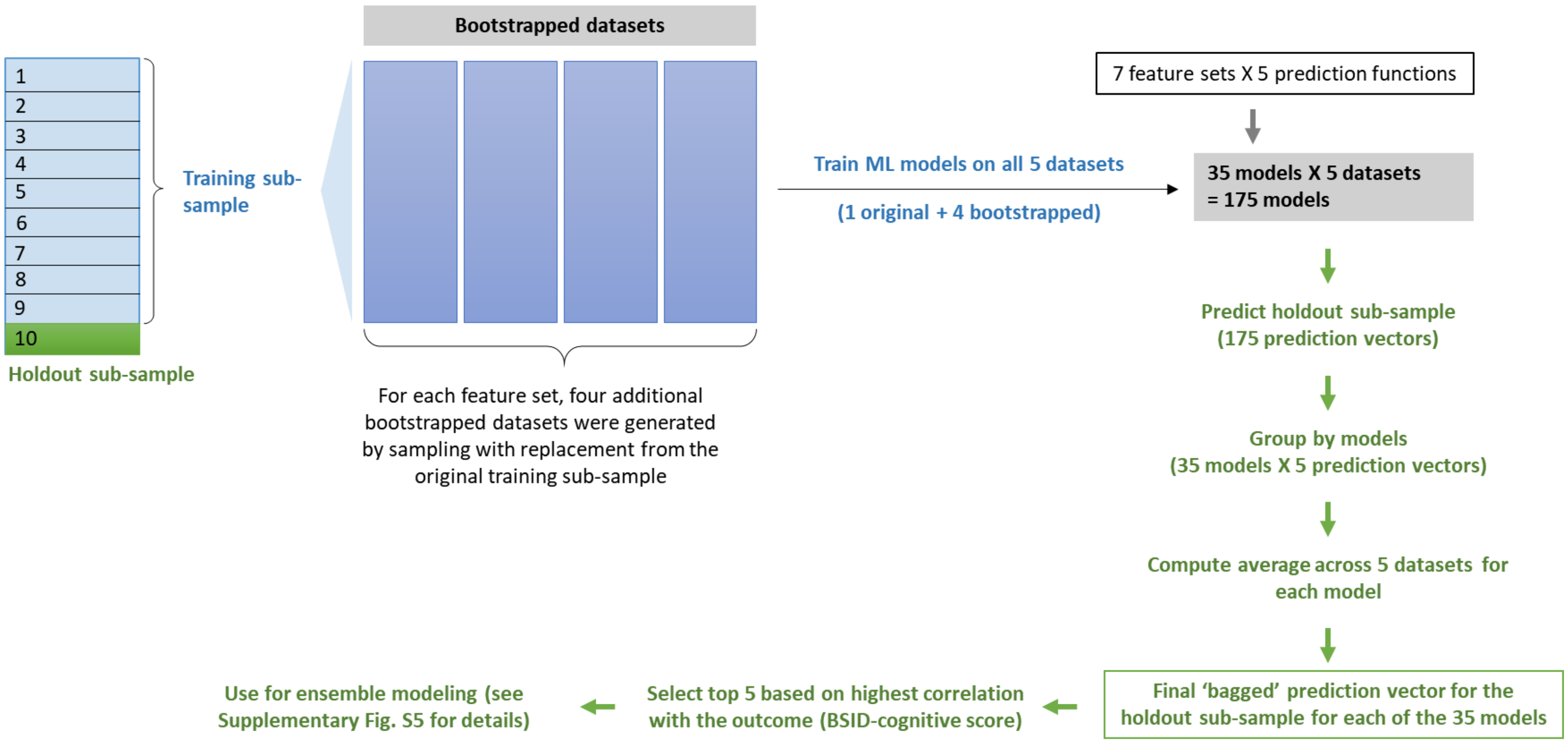

Supplementary Figure S4: Evaluating the bias-variance trade-off to determine the optimal number of top models to use for ensemble modelling

10-fold CV was repeated 5 times using 2-7 top models for ensemble modelling. The root-mean-squared error (RMSE) of the predictions for each run was plotted against the number of models used for ensembling. The boxplot demonstrates the mean (dotted line), median (solid line) and variability of the RMSE across 5 runs. 5 top models were selected for ensembling since it resulted in the least error and variance. Individual data points are plotted on the left of the boxplots for each condition.

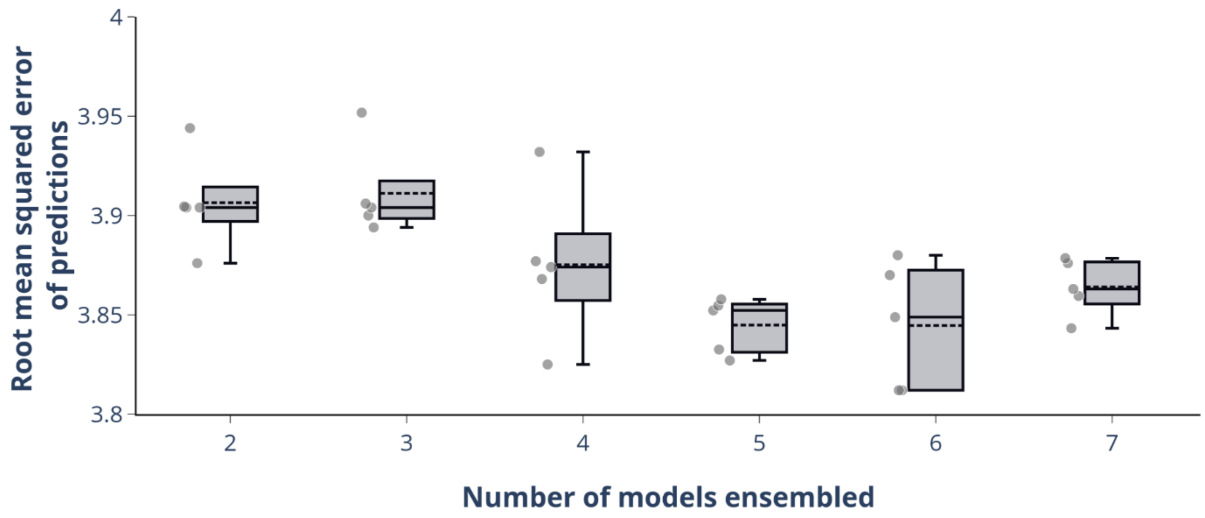

Supplementary Figure S5: Schematic of the ensemble modelling approach using stacking and weighted averaging used in this study

During each CV run, the top five 'bagged' predictions for the training subsample was used as input features to model the BSID-cognitive score using three stacking functions – linear regression, XGBoost and Random Forest, and then used to predict the holdout sample. These three stacked predictions were combined using 10 different weighted averaging schemes to generate ten 'ensembled' predictions for each child in the holdout sample. Once all ten repeats of the CV run were over, the ensembled prediction vector with the least root-mean-squared-error with respect to the outcome (BSID-cognitive score) was selected as the final predicted score (referred to in the manuscript as the DEEP score). Operations in the training sample are coded in blue while those in the holdout sample in green.

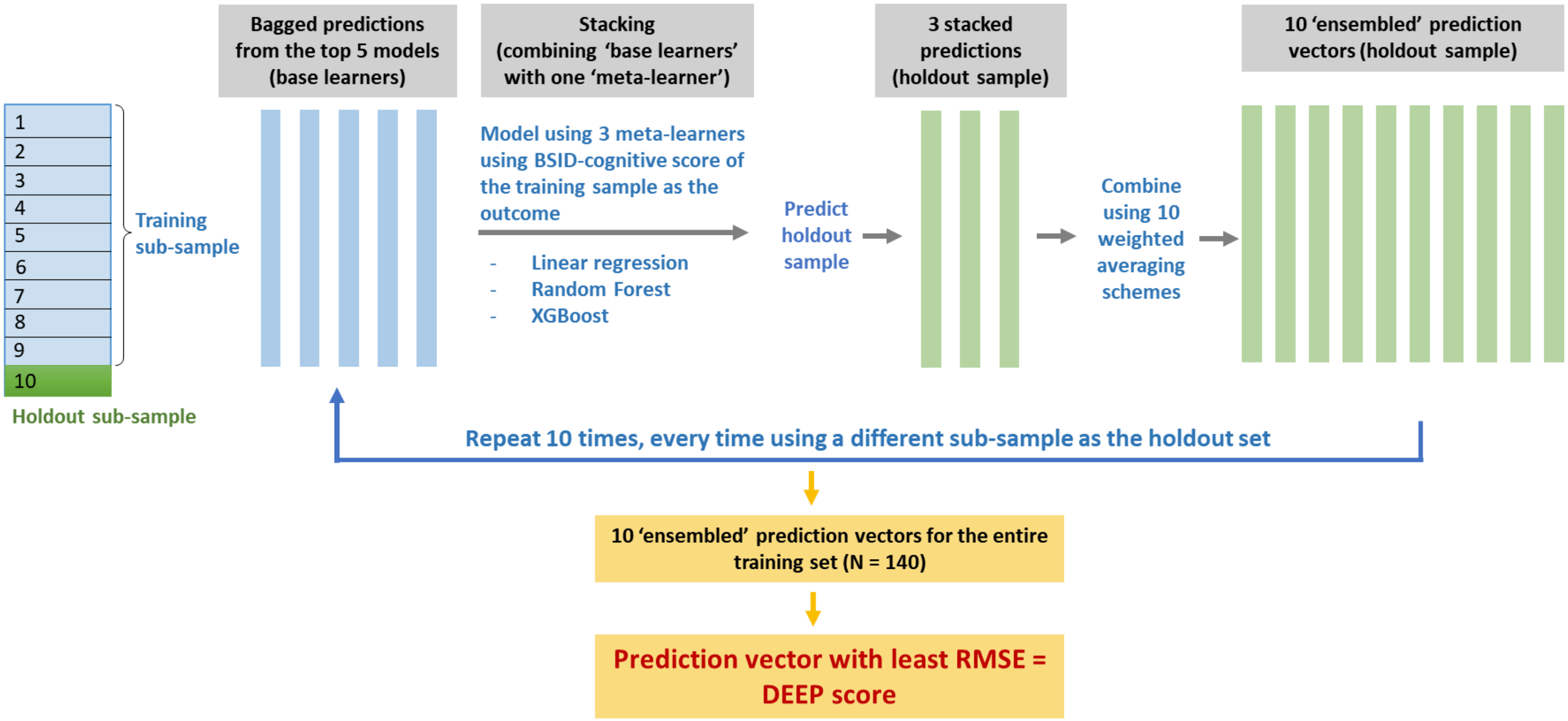

Supplementary Figure S6: Screenshots of games on DEEP, brief instructions on how to play the game, and important metrics collected in the backend to assess child performance

	Brief instructions	Backend metrics captured (with timestamps)
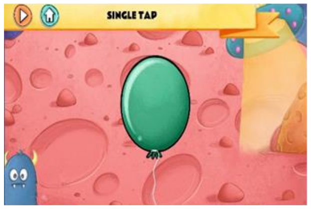	**Single tap**<br>Pop this balloon as fast as you can	- Correct taps (on the balloon)<br>- Background taps (outside the balloon)
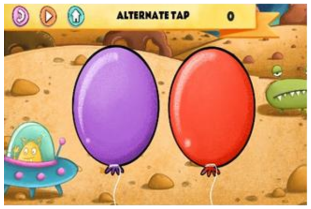	**Alternate tap**<br>Pop these balloons alternately as fast as you can	- Correct taps (Balloon highlighted for tapping)<br>- Background taps (outside the balloons)<br>- Incorrect taps (Balloon shaded out)
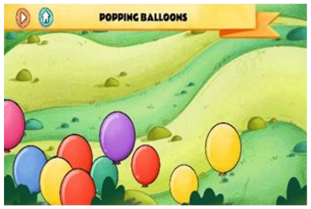	**Popping balloons**<br>Pop as many balloons as you can	- Correct taps (on the balloons)<br>- Background taps (outside the balloons)
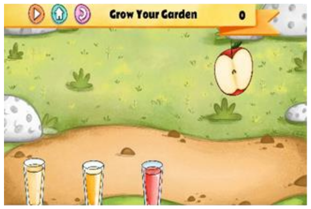	**Grow your garden**<br>Touch the apple, do not touch the bug	- Correct taps (on the apple)<br>- Background taps (outside the apple or bug)<br>- Incorrect taps (on the bug)
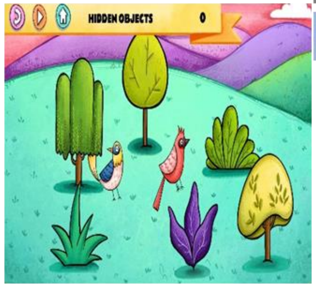	**Hidden objects**<br>Touch the hiding place of the birds	- Correct taps (hiding places)<br>- Background taps (outside all hiding places)<br>- Incorrect taps (Hiding places where no birds hid, or subsequent taps on places where the bird was found hiding
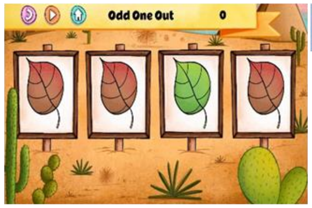	**Odd one out**<br>Touch the object which is different from the other 3	- Correct taps (on the object different from others)<br>- Background taps (outside the objects)<br>- Incorrect taps (on any of the three similar objects)
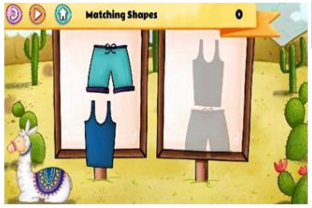	**Matching shapes**<br>Drag the objects to their matching shadows	- Correct drag (to the matching shadow)<br>- Incorrect drag (to any other location)
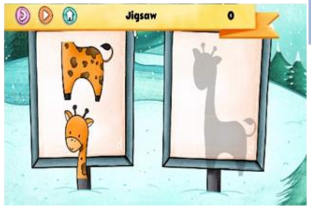	**Jigsaw**<br>Drag the parts of the animal to its shadow to make a whole	- Correct drag (to the correct location on the shadow)<br>- Incorrect drag (to any other location)
